# An Alternative to Institutional Segregation

**Published:** 1914-04-18

**Authors:** 


### THE PROBLEM OF THE ADVANCED CONSUMPTIVE.

### An Alternative to Institutional Segregation.

It is generally conceded that of all the manifold problems connected with the campaign against tuberculosis that of the advanced and infectious case is the most difficult to deal with. Both in the Metropolis and in the provinces this type of case is giving the authorities food for anxious thought. The altogether undue proportion of cases of phthisis occurring in small houses renders it urgently important that provision should be made for those persons suffering from the later stages of consumption, in order to obviate the serious danger that exists to the other inmates of the house. It has been pointed out that the number of cases of pulmonary tuberculosis per 1,000 of the population in different districts varies very closely in direct proportion to the number of one- and two-room tenements to be found in each district. It is largely through these advanced cases that infants die of tubercular meningitis, and tuberculous children are manufactured to fall a victim to re-awakened disease when their adult environment becomes adverse. We are not unmindful of the suggestion that a certain degree of immunity may be possessed by the more resistant ones who survive—but at what a cost! The question of immunity concerns rather the infection with bovine tuberculosis, and should not be considered in connection with the massive infection such as occurs from contact with human beings in advanced stages of the disease.

In what is known as the Edinburgh system the hospital for advanced cases is accorded a definite position. In actual practice very few schemes indeed have as yet provided any such accommodation, except on paper. For the poor the provision is narrowed down either to the general hospital or to the Poor-Law infirmary. The authorities of the former institution, in a perfectly defensible attitude, evince a great disinclination to accept the responsibilities in connection with the admission of such cases. The objections on the part of the friends to the segregation of their relatives in the wards of the infirmaries are perfectly natural, and in the present state of these wards, both as regards the staffing and the inmates, this reluctance can scarcely be condemned.

The provision of separate institutions, or, better still, the setting aside of special wards or blocks in existing hospitals for this class of case is a need which is gradually being recognised. But it must be expected that such accommodation will not be provided for some time to come. Meanwhile, the problem remains; and, indeed, it may be hoped that it is at its worst phase now, for ameliorative measures may be trusted as time goes on to reduce its dangers. Is there, then, no other expedient which may be tried? There exists a large class of poor consumptives whose social condition is above that of the infirmary class and who are sufficiently aware of the danger of harbouring infectious relatives to co-operate usefully with efforts made by dispensary and other workers to minimise this danger. To such people assistance might be given in the direction of providing extra accommodation in the shape of an additional room or, if space out of doors were available, of erecting a shelter for the sole use of the advanced infectious case. Such assistance would be far less expensive than the twenty-five or thirty shillings a week which is the cost of segregation in an institution. The objection that this method is open to abuse is, of course, one that cannot be ignored, but from the experience of case committees, working in connection with tuberculosis dispensaries, we judge that there are many cases in which such assistance could be justifiably and successfully given.

There is already at work a small army of district visitors, health visitors, and dispensary nurses, who are continually inspecting homes, so that a fairly strict eye could be kept on such families as are being assisted in this way. The type of family suitable for such help can usually be gauged accurately by those who know the people; and even if a strict isolation of the advanced patient were not kept, surely it would be easy to effect a great improvement over the conditions too often found, where an infectious patient sleeps not only in the same room but in the same bed with his wife and perhaps a child also.

We believe that, pending the provision of beds in separate institutions for advanced cases of tuberculosis of the lungs, such assistance as indicated above would not only repay itself in lessening the danger to children and others, but would form a salutary educative measure. The difficulty of selecting appropriate cases is one which can be overcome with the assistance of the mass of information accumulated by registrars of the Charity Organisation and other societies.

